# Surgical Treatment for Patent Ductus Arteriosus: Our Experience of 12 Years

**DOI:** 10.7759/cureus.14731

**Published:** 2021-04-28

**Authors:** Mehmet Tort, Münacettin Ceviz, Fehimcan Sevil, Necip Becit

**Affiliations:** 1 Cardiovascular Surgery, Ataturk University School of Medicine, Erzurum, TUR; 2 Cardiovascular Surgery, Afyonkarahisar Health Sciences University, Afyonkarahisar, TUR

**Keywords:** pda, patent ductus arteriosus, pulmonary hypertension, surgical closure, ligation of pda

## Abstract

Introduction

Patent ductus arteriosus (PDA) is a congenital heart disease that, if left untreated, can lead to pulmonary hypertension, congestive heart failure, and death. Here, we aimed to assess postoperative cardiac hemodynamic changes and surgical techniques, as well as early and late postoperative findings in surgically treated PDA patients.

Materials and methods

We retrospectively analyzed the data belonging to 126 patients whose PDA was surgically closed in our clinic from January 2001 to December 2012. With echocardiography being a standard in diagnosis and follow-up, angiography and computed tomography were also used in the presence of pulmonary hypertension and congenital heart disease, when needed. Postoperative data were compared between isolated PDA patients and those with congenital cardiac deformities.

Results

Evaluating the patients' pulmonary artery pressure (PAP), pulmonary hypertension was detected in 121 patients (96.0%). Preoperative PAP was significantly higher in PDA patients with congenital heart disease compared to the isolated PDA group (p<0.05). PAP decreased significantly in postoperative follow-up in both groups (p<0.05). However, this decrease was faster in the isolated PDA group than in patients with congenital heart disease and right-left shunt accompanying PDA (p<0.05). Regarding the correlation between ductus diameters and preoperative PAP, we found that as ductus diameter increased, PAP increased significantly (p<0.05).

Conclusions

In PDA patients, closing the ductus is necessary to prevent pulmonary and cardiac complications. Surgical closure remains one of the most effective methods for this, although there is little difference between surgical treatment methods in terms of mortality.

## Introduction

Patent ductus arteriosus (PDA) is one of the most common congenital heart defects, particularly in premature births, constituting 5-10% of all congenital heart diseases [1-2]. If left untreated, it can lead to pulmonary hypertension, congestive heart failure, and death [3]. Also, complications like bronchopulmonary dysplasia, necrotizing enterocolitis, and intraventricular hemorrhage are common, but these neonatal morbidities can be reduced significantly with early diagnosis and treatment [4]. Treatment varies based on the patient, but PDA can be closed by medical or surgical methods, including catheterization. In the current study, we evaluated surgical techniques performed on patients undergoing surgical PDA closure, preoperative and postoperative clinical findings, and early and late outcomes.

## Materials and methods

We retrospectively analyzed the data belonging to 126 patients whose PDA was surgically closed in our clinic from January 2001 to December 2012. All PDA patients whose diagnosed and surgically treated between the specified dates were included in the study. None of the patients whose PDA was surgically closed were excluded from the study.

Patients' demographic characteristics, symptoms, diagnostic methods, postoperative findings, surgical complications, and mortality rates were investigated. Infants born before the 37th gestational week were considered premature. Although echocardiography (ECHO) was used for preoperative imaging, interventional angiography and/or computed tomography were performed to evaluate pulmonary vascular resistance and other accompanying cardiac deformities in patients with severe pulmonary hypertension. Patients with pulmonary artery pressure (PAP) over 25 mm/Hg were evaluated as pulmonary hypertension (PH) [5]. ECHO was used during follow-up, and PAP, left atrium diameter, and ejection fraction were evaluated.

The patients were clinically categorized into three groups [6]. Those with a small PDA had presented as asymptomatic, had a systolic or continuous murmur, and their heart chambers and PAP were normal or slightly increased. The moderate PDA group had a continuous murmur and a significant volume load. The large PDA group had presented as symptomatic and showed progression to advanced pulmonary hypertension or aggressively to Eisenmenger syndrome.

Besides, patients were categorized into two other groups as isolated PDA and those with congenital heart disease. Preoperative data and first and sixth-month postoperative follow-up data were analyzed and the statistical significance was revealed.

Ethics committee

The study protocol adhered to the guidelines stipulated in the Declaration of Helsinki and was approved as an electronic medical record-based retrospective study by the Institutional Review Board of Ataturk University Faculty of Medicine, Department of Cardiovascular Surgery (24.05.2012 -No: B.30.2.ATA.0.01.00/39); as such, the requirement for obtaining informed consent from the patients prior to study participation was waived.

Statistical analysis

Continuous variables were expressed as mean ± standard deviation and categorical variables as percentages. Friedman Test was used to compare the changes of continuous variables at baseline, first, and sixth months. Data were analyzed using SPSS 15.0 for Windows program (SPSS Inc., Chicago, USA). P<0.05 value was accepted as the limit of significance.

## Results

Surgical PDA closure was performed on 126 patients over a period of 12 years. Of these patients, 66 (52.4%) were male and 60 (47.6%) were female. Regarding age distribution, the patients had a mean age of 5.4±0.7 years (range: 5 days and 47 years). Surgical closure was performed in 52 (41.3%) patients in the first three months after birth due to large PDA. 48 of these patients (38.0%) were premature infants born. The age distribution of the patients is given in Table 1. 

**Table 1:** Age distribution of the patients

Patient Age	Patient (n)	Percentage
0-3 months	52	41.3%
3-12 months	24	19%
1-5 years	18	14.3%
6-9 years	13	10.3%
10-19 years	10	7.9%
20-29 years	4	3.2%
30 years or above	5	4.0%
Total	126	100%

Forty-three (34.13%) of the patients were asymptomatic. Among the symptoms, dyspnoea was the most common in 22 (17.46%) patients, while 20 (15.87%) were hospitalized with a diagnosis of recurrent lung infections and then diagnosed with PDA. The most common finding in preoperative physical examinations was the characteristic PDA murmur. A total of 122 (96.8%) of the patients had isolated PDA murmur, 34 (27.0%) had accompanying hepatomegaly, and 27 (21.4%) had thrill. Other less common symptoms and clinical findings are summarized in Table 2. Considering preoperative chest radiographs, pulmonary conus was observed to be flattened in 53 patients (42%). Most patients (63.5%) showed no abnormal electrical activity in their electrocardiography (ECG) evaluation (Table 2).

**Table 2:** Clinical characteristics of the operated patients ASD: atrial septal defect, VSD: ventriküler septal defect, LV: left ventricle, RV: right ventricle, PDA: patent ductus arteriosus, ECG: electrocardiography

		n	%
Symptoms			
	Asymptomatic	43	34.1
	Dyspnoea	22	17.5
	Recurrent Lung Infections	20	15.9
	Respiratory Distress Syndrome	17	13.5
	Palpitation	15	11.9
	Growth retardation	13	10.3
	Cyanosis	11	8.7
Clinical Findings			
	Murmur of PDA	122	96.8
	Hepatomegaly	34	27.0
	Thrill	27	21.4
	Nonpalpable Femoral Pulse	7	5.6
X-Ray			
	Pulmonary Conus Flattening	53	42.0
	Increased Pulmonary Vascularity	35	27.8
	Normal X-Ray	38	30.2
ECG			
	Normal ECG	80	63.5
	LV hypertrophy	23	18.3
	Biventricular hypertrophy	13	10.3
	RV hypertrophy	8	6.3
	First degree AV Block	1	0.8
	Right bundle branch block	1	0.8
Additional cardiac pathology		58	46.0
	ASD	31	24.6
	ASD+ VSD	11	8.7
	VSD	4	3.2
	Coarctation of the aorta+ ASD	3	2.4
	Coarctation of the aorta+ VSD	2	1.6
	Coarctation of the aorta	2	1.6
	Others	5	3.9
Genetic Anomalies			
	Down syndrome	13	10.3
	Cleft palate deformity	3	2.38

Fifty-eight (46.0%) patients had accompanying congenital heart disease (CHD), while one had chronic renal failure and another had a four-month pregnancy. The most common accompanying cardiac pathology was atrial septal defect (ASD) in 31 (24.6%) patients, followed by ASD+ ventricular septal defect (VSD) in 11 (8.7%). Other less common pathologies accompanying PDA are given in Table 2. Regarding genetic anomalies accompanying PDA, Down Syndrome was the most frequent in 13 (10.3%) patients. Three (2.38%) patients had cleft palate malformation.

Echocardiography was the standard for all PDA patients in preoperative evaluation, but angiography was performed after ECHO in 33 (26.2%). ECHO, angiography, and computed tomography (CT) were used together in six patients.

**Figure 1:**
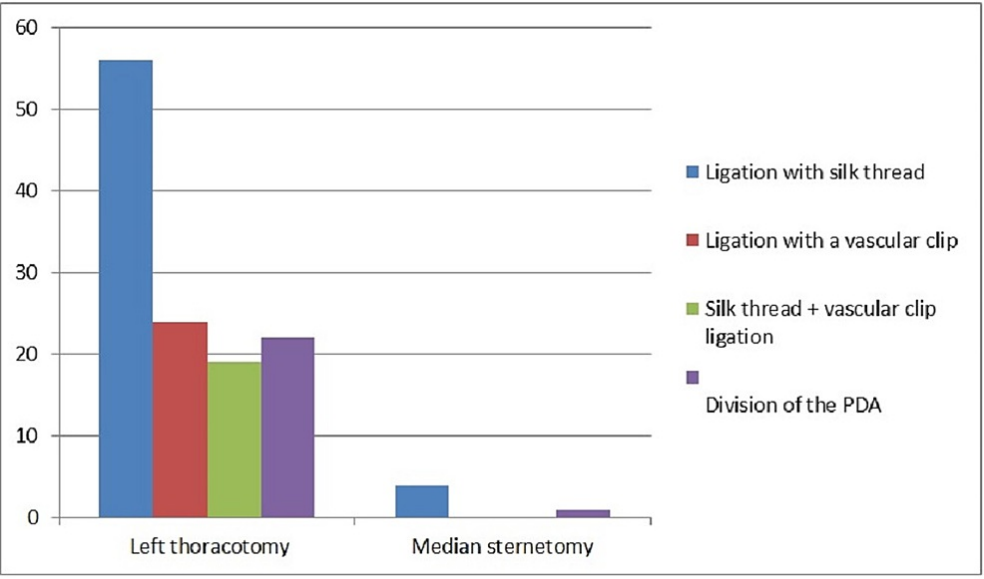
Surgical techniques applied to patients PDA: patent ductus arteriosus

Of the surgical procedures performed on our patients, 121 (96.0%) were done by the left posterolateral thoracotomy approach and no cardiopulmonary bypass (CPB) was needed. Ligation was performed in 99 (89.3%) of these patients. Out of these 99 patients, 56 (56.6%) were ligated with silk, 24 (24.2%) were provided PDA occlusion using hemoclips, and hemoclips and silk ligation were used together in 19 (19.2%). Fifteen of our patients (12.3%) were applied the division technique due to calcific and fragile ductus (Figure 1). In 5 of 121 patients (6.6%), PDA excision was done with coarctic aortic segment, along with end-to-end anastomosis of the aortic segments due to accompanying aortic coarctation (Figure 1).

**Figure 2:**
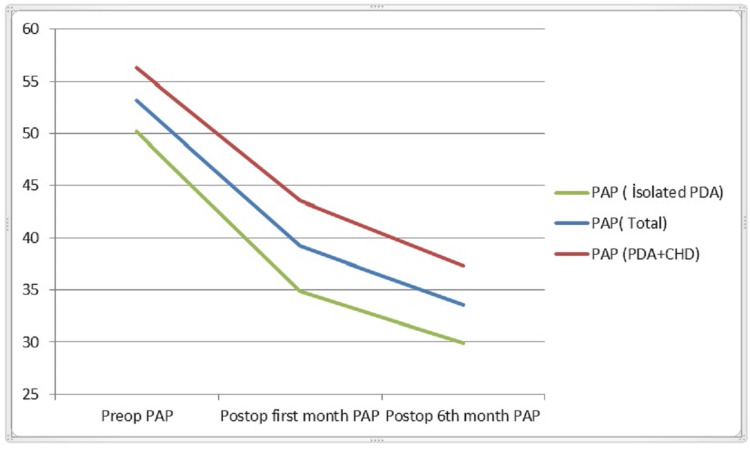
Change of PAP in the group with and without congenital heart disease PAP: pulmonary artery pressure, PDA: patent ductus arteriosus, CHD: congenital heart disease, Preop: preoperative, Postop: postoperative

Evaluating the patients' PAP, pulmonary hypertension was detected in 121 patients (96.0%). Mean preoperative PAP was 58.1±21.88 mm/hg. A total of 122 out of 126 patients who were followed with no mortality had a mean preoperative PAP of 53.2±21.5 mm/hg, a mean postoperative first month PAP of 39.2±16.1 mm/hg, and a mean postoperative sixth month PAP of 33.6±13.2 mm/hg. Preoperative PAP was significantly higher in PDA patients with congenital heart disease compared to the isolated PDA group (p<0.05). PAP decreased significantly in postoperative follow-up in both groups (p<0.05). However, this decrease was found to be faster in the isolated PDA group than in those with congenital heart disease and right-left shunt accompanying PDA (p<0.05) (Figure 2).

We observed small PDA in seven (5.6%) patients, moderate PDA in 22 (17.5%), and large PDA in 97 (76.9%). Regarding the correlation between ductus diameters and preoperative PAP, we found that as ductus diameter increased, PAP increased significantly (p<0.05). Based on ductus diameters, the PAP values of all three groups decreased in the postoperative first and sixth months, although this decrease was significant in the moderate and large PDA groups (p<0.05) (Table 3). In the postoperative first and sixth-month ECHO examinations of our patients, left atrium diameters were found to have decreased compared to preoperative data, with statistical significance in patients under 5 years of age (p<0.05) (Table 3). There was no significant difference between the groups in terms of preoperative and postoperative ejection fraction (EF).

**Table 3:** Ejection fraction (EF), left atrium (LA) diameter (mm), and pulmonary artery pressure (PAP) values (mm/Hg) changes before and after the operation CHD: congenital heart disease, PDA: patent ductus arteriosus

	Pre-operative	Post-operative first month	Post-operative sixth month	P-value
PAP (All Patients)	53.2 (±21.5)	39.2 (±16.1)	33.6 (±13.2)	<0.05
According to whether there is an accompanying heart disease				
Isolated PDA (PAP)	50.2 (±21.4)	34.9 (±11.9)	29.9 (±9.2)	<0.05
PDA + CHD (PAP)	56.3 (±21.4)	43.6 (±18.7)	37.3 (±15.7)	<0.05
According to the clinical classification				
Small PDA (PAP)	25.3 (±5.3)	22.8(±6.1)	21.3 (±7.3)	0.432
Medium PDA (PAP)	42.8 (±14.3)	32.9 (±10.6)	28.5 (±7.1)	<0.05
Large PDA (PAP)	58.5 (±21.5)	42.1 (±17.1)	35.8(±14.3)	<0.05
LA Diameters				
LA diameter in patients under 5 years of age	18.6 (±8.3)	16 (±5.4)	14.4 (±5.1)	<0.05
LA diameter in all patients	22.3 (±11.2)	20.1 (±9.5)	19.3 (±8.1)	0.137
EF (All patients)	71 (±8)	70 (±9)	72 (±8)	0.321

In five (3.4%) patients, accompanying cardiac pathologies were repaired under CPB with median sternotomy and additional PDA closure was done. Of these five patients, four were applied ligation with silk. For the remaining one patient, PDA closure was achieved by the division technique. None of our patients had a total cardiopulmonary arrest.

The mean length of hospital stay was 5 days (3-23 days). The mean number of postoperative residual shunting was 5 (3.9%). One of our patients underwent revision on the postoperative eighth hour due to thoracic hematoma, although no bleeding focus was observed. None of our patients had any complications like infection, aneurysm formation, nerve injury, or chylothorax. Mortality occurred in four patients (3.1%), all of whom had congenital heart disease.

## Discussion

Despite the recent increase in PDA closured with catheterization, surgical PDA closure along with medical treatment has been the gold standard for long [2]. Medical treatment still involves risks such as necrotizing enterocolitis and intracranial bleeding [7], although there are certain restrictive factors depending on the type of percutaneous occlusal devices used for PDA closure in infants with low weight and in patients for whom ductus anatomy is appropriate [2,8]. Surgical PDA closure, on the other hand, can be performed on all patients regardless of age or ductus anatomy.

Patent ductus arteriosus is a common congenital deformity that if left untreated can lead to pulmonary hypertension and Eisenmenger syndrome [7]. It has an increasing frequency in premature births, reportedly up to 70% in deliveries before the 28th gestational week [9]. Here, most of our patients (48/52) were aged 0-3 months and were premature infants with low birth weight (<2000 g). Given the incidence of PDA, it is twice more likely to occur in girls than in boys [10]. In the present study, the female to male ratio was nearly equal. Yet, this lack of significant difference is explained by the inclusion of only PDA cases with surgical intervention, and not all cases with a diagnosis of PDA. There are other studies with similar sex ratios [11].

Symptoms can range from a completely asymptomatic clinical course to congestive heart failure. The most important factors that determine the clinical course are the ductus arteriosus diameter and the amount of shunting [12]. Most of our patients (34.1%) presented as asymptomatic due to their ductus diameters and early diagnosis. The most common physical examination finding is continuous murmur proportional to the shunt flow through the duct. Thus, in patients with increased pulmonary artery pressure and decreased shunt from the aorta to the pulmonary artery, the murmur may not be heard even when the PDA is large [13]. In this study, continuous PDA murmur was detected in 96.7% of the patients and 4 (3.2%) had severe pulmonary hypertension and large PDA due to catheterization, but low shunt flow even though no cardiac murmur was detected.

Echocardiography is the gold standard for early diagnosis of ductus arteriosus and for determining its hemodynamic significance, particularly in premature infants. The direction of shunting can be monitored using color Doppler echocardiography [14-15]. However, angiographic catheterization allows pulmonary reactivity tests in patients with high ductus diameter, pulmonary vascular resistance, and pulmonary artery pressure [10,13]. In the presence of other congenital heart diseases accompanying PDA, cardiovascular magnetic resonance (CMR), cardiovascular computed tomography (CCT), and stress imaging can be performed for a better cardiac evaluation [16]. Regarding the preoperative evaluation of the patients surgically treated in our clinic, ECHO was sufficient in 87 (69%) patients. Other imaging tests were performed in patients with congenital heart disease accompanying PDA. The remaining patients underwent angiographic imaging in addition to ECHO, 33 (26.2%) due to other cardiac pathologies or high pulmonary hypertension and 6 (4.8%) due to other accompanying diseases (interrupted aorta, aortic coarctation, etc.) ECHO, CCT, and catheterization were performed. Here, postoperative follow-up was done by echocardiography in accordance with the literature [16]. Other imaging methods were not needed.

Patent ductus arteriosus closure begins in the first few hours after birth and is anatomically completed in 2-3 weeks [8]. There is yet discussion on which patients require PDA closure, when it should be closed, and the methods of closure [17-19]. PDA closure is recommended for cases with left atrium or left ventricular enlargement, a clear left-to-right shunt flow through the ductus, and a pulmonary artery pressure (PAP) to systemic arterial pressure (SAP) rate of less than 0.5. In patients with a clear left-to-right shunt, closure should still be considered when the PAP/SAP rate is greater than 0.5, but pulmonary vascular resistance is below 2/3 of the systemic vascular resistance. However, PDA closure is not recommended in cases where pulmonary vascular resistance is above 2/3 of the systemic vascular resistance or where shunt flow is right-to-left [10]. Here, a pulmonary reactivity test with adenosine was done to assess the pulmonary capillary bed and postoperative prognosis in four cases with a PAP/SAP rate of >0.5, and operation was decided when PAP was found to decrease. Also, these patients underwent occlusion tests and their ductus were successfully closed after PAP was decreased. While PAP is not affected too much in patients with small ductus, it remains a risk factor for the development of endarteritis [20]. In our clinic, closure was done as quickly as possible to minimize the development of complications in symptomatic PDA patients and asymptomatic cases with a severe shunt. Hemodynamically stable cases with a small PDA and low shunt were closed before the age of 1 year if they were not closed after medical treatment.

For patients with moderate to large PDA, increased pulmonary pressure can lead to increased pulmonary vascular resistance and congestive heart failure, secondary lung complications, and irreversible heart and lung pathologies, which may result in Eisenmenger Syndrome [21]. We found a directly proportional correlation between ductus size and PAP (p<0.05). After ductus arteriosus occlusion, the shunt from the left system to the right system disappears. This results in decreased PAP [12]. In the current study, there was a statistically significant decrease in PAP after ductus closure, which was mostly observed in patients with moderate to large ductus diameters. This decrease in PAP was found to have continued in the sixth-month follow-up. This shows similarity to the findings of previous research on patients with pulmonary arterial hypertension (PAH), regardless of the method used for ductus closure [22].

Patent ductus arteriosus patients have an increased left ventricle (LV) volume load and workload depending on the amount of shunt flow from the aorta to the pulmonary artery. Secondary to this, there may be left atrium and left ventricle enlargement and myocardial hypertrophy over time. Some studies have reported a temporary decrease in left ventricular ejection fraction in the early period after PDA closure. This was found to be associated with a sudden decrease in preload and an increase in afterload in the left ventricle, which is overloaded before closure, particularly after PDA closure with high shunt flow or in cases with high PAP [22-23]. Yet, we did not encounter this complication in our study. We measured left atrium (LA) dimensions after ductus occlusion using ECHO at the first and sixth postoperative months and found a statistically significant decrease in LA size, particularly in patients aged 0-5 years. We can conclude from this that atrial dilatation normalizes more slowly and may be permanent in chronic PDA cases compared to patients treated earlier.

After PDA ligation, the most common complications are vocal cord paralysis, bronchopulmonary dysplasia, retinopathy of prematurity, chylothorax, pneumothorax, bleeding, diaphragmatic paralysis, and cardiopulmonary insufficiency and infections [24]. Residual flow after PDA ligation may be observed in 1.5-6% of cases [25-26]. A residual shunt is not observed in cases with ductus division, although this technique requires more time and effort. On the other hand, ductus ligation has a lower complication risk, is easier to perform, and has a shorter duration of surgery [7]. In our sample, a residual shunt was observed in five (3.9%) patients. In four of these patients, the residual shunt was closed by catheterization. A residual shunt was also observed in one patient in the first postoperative year and was reoperated for division. One of our patients was taken to revision due to the detection of a thoracic hematoma 8 hours after the operation, but no bleeding focus was detected. No infection, aneurysm formation, nerve injury, or chylothorax was observed in any of our patients. In our clinic, the total number of mortalities after PDA surgery was four (3.1%). There was a history of premature birth in two patients (50.0%) who were ligated with left posterolateral thoracotomy and developed mortality. The first of these patients whom died due to mechanical ventilator complications on the first postoperative day and the other due to aspiration pneumonia during pediatric third-level ICU follow-ups on the fifth postoperative day. Both of these two patients had a congenital heart disease accompanying PDA (ASD in the first, VSD in the second) and preoperative PAP values of 31 and 98 mm/Hg, respectively. Two other patients resulted in exitus due to low cardiac output and multiorgan failure caused by open heart surgery, who had PDA occlusion and accompanying Fallot tetralogy and VSD + aortic coarctation. None of our patients who were operated on for isolated PDA died. In the literature, mortality after PDA ligation has been reported as 4.3% by Kang et al. [27] and 6.5% in the 2817-case series of Reese et al. [11]. There are other studies where no mortality was reported after isolated PDA ligation [26]. In this respect, our findings were similar to the literature and no mortality was observed after surgery only in patients with isolated PDA. Of the four patients with mortality, two had a history of premature birth. If the mortality rate between premature and term birth is analyzed, the mortality rate in premature babies is 4.1%, while this rate is 2.5% in those with a term birth.

## Conclusions

In PDA patients, closing the ductus is necessary to prevent pulmonary and cardiac complications. Surgical closure remains one of the most effective methods for this, although there is little difference between surgical treatment methods in terms of mortality. Ductus closure by the ligation technique can be considered an advantage for being easier, more reliable, and having no difference in terms of mortality or morbidity.
